# Lower HLA-G levels in extravillous trophoblasts of human term placenta in gestational diabetes mellitus than in normal controls

**DOI:** 10.1007/s00418-022-02163-4

**Published:** 2022-12-20

**Authors:** Julia Knabl, Rebecca Hüttenbrenner, Sven Mahner, Franz Kainer, Gernot Desoye, Udo Jeschke

**Affiliations:** 1 Department of Gynecology and Obstetrics, University Hospital–Ludwig Maximilian University (LMU) Munich, Marchioninistr. 15, 81377 Munich, Germany; 2 Department of Obstetrics, Klinik Hallerwiese, 90419 Nuremberg, Germany; 3 Department of Obstetrics and Gynecology, Medical University Graz, Auenbruggerplatz 14, 8036 Graz, Austria; 4 Department of Obstetrics and Gynecology, University Hospital Augsburg, Stenglinstr.2, 86156 Augsburg, Germany

**Keywords:** Pregnancy, Diabetes, HLA-G, Trophoblast, Sex, Human

## Abstract

The non-classical human leucocyte antigen (HLA) class I molecule HLA-G is widely known to play a major role in feto-maternal tolerance. We tested the hypothesis that HLA-G expression is altered in placentas of women with gestational diabetes mellitus (GDM) in a specific pattern that depends on fetal sex. HLA-G expression was analysed in a total of 80 placentas (40 placentas from women with GDM and 40 healthy controls) by immunohistochemistry using the semi-quantitative immunoreactive score (IRS). Double immunofluorescence staining identified the cells expressing HLA-G in the decidua and allowed evaluation of the expression pattern. We found a significant (*p* < 0.001) reduction of HLA-G expression in extravillous cytotrophoblasts (EVTs) in the placentas of women with GDM as compared to the healthy controls and were able to demonstrate that this downregulation was not due to a loss of cell number, but to a loss of expression intensity. A special change in the cell pattern of EVTs was observed, with these cells showing an obvious decrease in HLA-G expression on their cell surface. No significant differences according to fetal sex were found. These data show a possible association between decreased HLA-G expression and presence of GDM and provide new insights into altered placental function in women with GDM*.*

## Introduction

During pregnancy, the semi-allogenic fetus needs to induce maternal immunotolerance in order to prevent its rejection. An important role in this special immune suppression system in pregnancy is attributed to the non-classical major histocompatibility complex (MHC) class I molecule human leucocyte antigen G (HLC-G) (Hunt et al. 2005). HLA-G is expressed in placental tissue invading the maternal uterine decidua during implantation. Its highest expression levels are recognized in extravillous cytotrophoblast cells (EVTs) with little, if any, expression on the syncytiotrophoblast (SCT) (Le Bouteiller and Lenfant 1996; O'Callaghan and Bell 1998). EVTs are positioned close to maternal immune cells at the materno-fetal interface and are well known for their contribution to maternal immunotolerance. EVTs do not express the classical MHC class I molecules HLA-A and -B. Consequently, the invasive trophoblast is not recognized as non-self by the maternal immune system (Bainbridge et al. 1999, 2000), resulting in the inability to induce immune responses.

Several HLA-G isoforms exist as a result of alternative splicing (Hunt et al. 2005). These mRNA variants encode one full-length isoform (HLA-G1), three short membrane-bound isoforms (HLA-G2, G3, G4) and two soluble isoforms (HLA-G5, G6). Current evidence suggests that only the full-length, cell surface-bound isoform HLA-G1 and the soluble HLA-G5 and -G6 are biologically active. Among these isoforms, HLA-G1 is expressed on EVTs, and although it may act as a classical HLA class I molecule, it also does not induce the maternal immune response (Bainbridge et al. 1999). The soluble isoforms HLA-G5 and G6 are not synthesized or secreted by human trophoblasts in detectable amounts (Blaschitz et al. 2005).

The HLA-G gene almost completely lacks polymorphisms and, therefore, there is little variation between individuals. However, HLA-G expression levels can be altered by disorders of reproduction; for example, lower HLA-G expression levels were found in women with early pregnancy failure, such as miscarriage and recurrent abortion (Moreau et al. 2009; Quach et al. 2014; Ferreira et al. 2016). In two other studies, at the time of delivery, the placentas of women with pre-eclampsia were found to have a significantly reduced expression of HLA-G compared to those of a healthy control group (Goldman-Wohl et al. 2000; Yie et al. 2004). HLA-G has been implicated in the complex network governing EVT invasion through an interaction with uterine natural killer (NK) cells. Several inhibitory receptors present on NK cells have been shown to bind to HLA-G, thereby promoting upregulation of inhibitory receptors on NK cells and CD4^+^ T cells (LeMaoult et al. 2005). It remains elusive whether these changes in expression with miscarriage and pre-eclampsia influences trophoblast invasion and placentation or whether they are just a consequence of disturbed blood perfusion and placental hypoxia (Rouas-Freiss et al. 2021). Intriguingly, HLA-G expression has also been detected in tumor lesions, where it may facilitate immune evasion (Rouas-Freiss et al. 1999; Wiendl et al. 2002; Ferreira et al. 2016).

The restrictive expression pattern of HLA-G expression with a very strong expression in invasive EVTs of the placenta (Kovats et al. 1990; Hackmon et al. 2017) makes it a widely accepted marker for the identification of EVT cells in immunohistochochemical staining procedures. The mRNA or protein levels of EVT cells can also serve as a normalization parameter in analyses of total first trimester trophoblast cell preparations. These cell populations contain a mixed population of villous and extravillous cytotrophoblasts as well as of syncytiotrophoblast fragments (Blaschitz et al. 2000), with variable proportions of each cell type in different preparations. Although purification steps can be added that enable the separation of these preparations into villous and extravillous populations, this comes at the expense of yield (Majali-Martinez et al. 2018) and, therefore, purification is not a commonly used step. As an alternative option, cell type-specific expression levels of any gene can be corrected for potential variation in EVT proportion in each cell isolation.

Gestational diabetes mellitus (GDM) is defined as glucose intolerance that is first diagnosed during pregnancy. Between 5 and 15% of all pregnancies are currently affected by GDM (McIntyre et al. 2019). The risk for a woman to develop GDM is higher when she carries a male fetus (Jaskolka et al. 2015). A further risk contributing to the worldwide increasing GDM prevalence is maternal obesity (Ruchat et al. 2013). Due to a pronounced peripheral insulin resistance, women fail to maintain normoglycemia (Metzger et al. 2007). GDM is associated with short- and long-term complications for the offspring. The short-term consequences included perinatal complications due to high birth weight and fetal hyperinsulinemia (Schwartz 1990; Hawdon 2011), and the long-term consequences include a higher risk to develop obesity, metabolic syndrome and type 2 diabetes in later life (Barnes-Powell 2007). GDM induces a proinflammatory environment in the placenta, as reflected by a prominent increase in markers and mediators of inflammation (Radaelli et al. 2003).

While alterations in HLA-G have been reported in women with pre-eclampsia at the end of their pregnancy (Goldman-Wohl et al. 2000; Yie et al. 2004), a potential effect of derangement of the maternal glucose-insulin axis, as in GDM, on HLA-G has not been investigated yet. Therefore, the aim of the present study was to localize HLA-G in the human placenta at full-term pregnancy and to quantify potential changes associated with GDM. We included villous tissue and decidua in the study. Sex-specific differences are common in placental function and pregnancy disorders, but it is as yet unknown whether also HLA-G expression shows sexual dichotomy.

## Materials and methods

### Study cohort

Following approval of the study design by the LMU Ethics Committee (approval number 337-06; approval date: 26 January 2010), 40 expectant women with GDM (GDM group) and 40 healthy expectant women (control group) were chosen to participate after written informed consent had been obtained. To be included in the study, all participants had undergone an oral glucose tolerance test (oGTT) between week 24 and 28 of their pregnancy (Carpenter and Coustan 1982). The diagnosis of GDM was based on the criteria of the German Society for Diabetes Mellitus, which are two measurements above accepted limits: fasting glucose > 90 mg/dL (5 mmol/L), at 1 h > 180 mg/dL (10 mmol/L) and at 2 h > 155 mg/dL (8.6 mmol/L). All women with GDM were managed with insulin and showed a mean glycated hemoglobin (HbA1c) of 5.8 ± 0.4%. A total of 75% of the patients had achieved good glucose control according to their mean blood glucose (≤ 100 mg/dL [5.6 mmol/L]). Detailed clinical and perinatal data on the study group have been published (Knabl et al. 2015a, b). Clinical data on the present study cohort are shown in Table 1 and stratified by fetal sex in Table 2. Fetal sex was balanced in the GDM and control group.

**Table 1:** Clinical details of the patients with gestational diabetes mellitus and of the normal healthy control group

Characteristics	GDM group (*n* = 40)	Healthy control group (*n* = 40)	*p*-value
Maternal age (years)	32.82 ± 4.56	31.15 ± 6.10	ns
Maternal BMI (pre-pregnancy, based on mother’s passport) (kg/m^2^)	28.1 ± 6.96	23.4 ± 6.21	< 0.0001*
Median gravidity (IQR)	2 (1.9)	2 (1)	ns
Median parity (IQR)	1 (1.4)	1 (1)	ns
Gestational age at delivery (weeks)	39.9 ± 1.3	39.8 ± 1.4	ns
With labour (%)	85	85	ns
Mean HbA1c %	5.8 ± 0.4	n.a	
Birth weight (g)	3611 ± 536	3317 ± 502	0.006*
Umbilical artery pH	7.30 ± 0.08	7.29 ± 0.09	ns
Median 5 min APGAR^a^ score (minimum; maximum)	10 (8; 10)	10 (8; 10)	ns
Sex of neonate (female/male)	20/20	20/20	

If not indicated otherwise, data are presented as the mean ± standard deviation (SD) or as the median with minimum and maximum in parentheses, as appropriate

*Significantly significant difference between groups, using the Mann–Whitney *U*-test to compare clinical outcome data of the two groups

*BMI* Body mass index,* GDM* gestational diabetes mellitus,* HbA1c* glycated hemoglobin,* IQR *interquartile range, *n.a.* data not available *ns* not significant

^a^APGAR score is a method to quickly summarize the health of newborn children (Appearance, Pulse, Grimace, Activity, Respiration)

**Table 2:** Clinical details of the patients with gestational diabetes mellitus and of the normal control group stratified by fetal sex

Characteristics	GDM group		Healthy control group		*p*-value
	Male (*n* = 20)	Female (*n* = 20)	Male (*n* = 20)	Female (*n* = 20)	
Maternal age (years)	31.5 ± 4.1	33.2 ± 5.33	30.3 ± 6.11	32.0 ± 6.13	ns
Maternal BMI (pre-pregnancy) (kg/m^2^)	29.4 ± 8.03	27.0 ± 4.73	21.9 ± 3.97	25.0 ± 7.90	< 0.001*
Gestational age at delivery (weeks)	39.7 ± 1.30	39.8 ± 1.40	39.8 ± 1.54	39.8 ± 1.16	ns
Median gravidity (IQR)	2 (1.75)	2 (2)	2 (1)	2 (1)	ns
Median parity (IQR)	2 (1.75)	1 (1)	1 (1)	2 (1)	ns
Mean HbA1c (%)	5.9 ± 0.4	5.7 ± 0.3	n. a	n. a	ns
With labour (%)	75	95	85	85	ns
Birth weight (g)	3662 ± 562	3636 ± 661	3340 ± 568	3294 ± 440	< 0.05*
Umbilical artery pH	7.30 ± 0.07	7.30 ± 0.10	7.30 ± 0.10	7.30 ± 0.08	ns
Median 5 min APGAR (minimum; maximum)	10 (9; 10)	10 (8; 10)	10 (8; 10)	10 (8; 10)	ns

If not indicated otherwise, data are presented as the mean ± SD or as the median with minimum and maximum in parentheses, as appropriate

*Statistically significant difference between groups: GDM group vs. control group including fetal sex, using the Kruskal–Wallis’s test to compare clinical outcome data of the four groups

### Tissue samples

Placentas were obtained within 5 min after birth. Tissue samples (2 × 2 × 2 cm^3^) were dissected from a central cotyledon of the placentas. The sampled tissues comprised decidua, villous tissue and amniotic epithelium. Macroscopically, the placentas were sufficiently supplied with blood; areas with signs of calcification, bleeding or ischemia were avoided. The tissue samples were fixed in 4% buffered formalin solution for 24 h and then embedded in paraffin for long-term storage.

### Immunohistochemistry

#### Staining and semi-quantification

Formalin-fixed paraffin-embedded, 3-µm-thick sections were deparaffinized in xylol, rehydrated in a descending ethanol gradient and subjected to epitope retrieval in a pressure cooker using sodium citrate buffer (pH 6.0), following which the sections were returned to room temperature and the endogenous peroxidase activity of the tissue was blocked with 3% H_2_O_2_ in methanol for 20 min. Non-specific binding of the primary antibodies was blocked by using the appropriate blocking solution (Table 3), followed by incubation with the primary antibodies. Salient features of the antibodies used are presented in Table 3. For staining, HLA-G antibody 4H84, which recognizes all HLA-G isoforms through an epitope located on the alpha-1 domain of HLA-G, was used. Immunoreactivity was detected by using the Vectastain Elite ABC-Kit (Vector Laboratories, Burlingame, CA, USA) according to the manufacturer’s protocol. As the final steps, substrate and chromogen (3,3′-diaminobenzidine [DAB]; Dako, Glostrup, Denmark) were added to the slides, which were then counterstained with Mayer’s acidic hematoxylin and covered with cover slips.

**Table 3:** Antibodies and dilutions used for immunohistochemistry and double immunofluorescence

Antibody	Dilution	Incubation	Manufacturer	Blocking solution	Blocking condition
Ck-7 (rabbit polyclonal)	1:100	Overnight	Santa Cruz Biotechnology (Dallus, TX, USA)	Ultra V Block	15 min
HLA-G clone MEM-6/9 (mouse IgG)	1:50	Overnight	AbD Serotec (Bio-Rad Laboratories, Hercules, CA, USA)	Ultra V Block	15 min
HLA-G clone 4H84 (Mouse IgG)	1:100 in power block; onefold dilution	Overnight	Novus Biologicals LLC (Centennial, CO, USA)	Power Block 1× solution	3 min
Negative control super sensitive mouse antibody	Ready to use	30 min room temperature	BioGenex Laboratories (Freemont, CA, USA)	Power Block 1× solution	3 min

*HLA-G* Human leucocyte antigen G,* IgG* immunoglobulin G

Placental tissue from women in their first trimester (8–12 weeks) of pregnancy was used for positive and negative controls. Primary anti-HLA-G antibodies were replaced by a ready-to-use commercial negative control (BioGenex Mouse Super Sensitive™ Negative Control [normal mouse IgG in phosphate-buffered saline (PBS) with carrier protein and preservative]; BioGenex Laboratories, Centennial, CO, USA; catalog no. HK119-7 M) for 30 min at room temperature according to manufacturer’s protocol.

The signals were semi-quantified using the semi-quantitative immunoreactivity score (IRS). Two independent examiners, blinded to group allocation of the sample, graded the optical staining intensity and the proportion of stained cells until consensus was reached. Grades for the staining intensity ranged from 0 to 3, with 0 indicating no signals/staining; 1, weak signals/staining; 2, moderate signals/staining; 3, strong signals/staining; those for the proportion of stained cells ranged from 0 to 4, with 0 indicating no stained cells; 1, ≤ 10% of the cells were stained; 2, 11–50% of the cells were stained; 3, 51–80% of the cells were stained; 4, ≥ 80% of the cells were stained. The IRS was calculated by multiplying the optical staining intensity by the percentage of stained cells.

The images were analysed under a light microscope (Immunohistochemistry Type 307–148.001 512 686; Leitz Microsystems, Wetzlar, Germany) equipped with a video camera (JVC CCD Color Video Kamera TK-C1380E C-Mount, Yokohama, Japan). For image acquisition, the software package Discuss version 4,602,017-#233 (Carl Horst Hilgers technical office, Königswinter, Germany) was used. Image bit depth was 24 mm, and time and space resolution data were 760 + 574 pixels.

#### Identification of cells expressing HLA-G in the decidua with double immunofluorescence

Double immunofluorescence staining was performed on decidua of from pregnant women with GDM and controls with the aim to identify the cell types expressing HLA-G. Placental sections were first deparaffinized in xylol for 20 min and then cleaned in ethanol, following which they were incubated in ethanol/methanol for 20 min. The slides were then rehydrated in an alcohol gradient and subsequently placed in a pressure cooker with sodium citrate for 5 min (pH 6.0). For fixation, sections were incubated at room temperature with acetone for 5 min, rinsed with PBS and then blocked with ultra-V blocking solution (LabVision Corporation, Freemont, CA, USA) for 15 min. The slides were then incubated with polyclonal rabbit CK-7 IgG to identify trophoblasts (Table 3) and subsequently incubated with monoclonal anti-HLA-G antibody overnight. Mouse anti-human HLA-G antibody, clone MEM-G/9, specifically recognizes surface-expressed native HLA-G1 when associated with beta 2 microglobulin, but not does recognize the isoforms HLA-G2, -G3 and -G4. The sections were then incubated with the secondary antibodies. The slides were incubated with the Cy-3-labelled goat-anti-rabbit IgG antibody (Dianova, Hamburg, Germany), which was diluted 1:500, and the Cy-2-labeled goat-anti-mouse IgG antibody, diluted 1:100. Next, the slides were embedded in DAPI containing mounting buffer (Vector Laboratories, Newark, CA, USA). Finally, the slides were analysed under a fluorescent Axioscop photomicroscope (Carl Zeiss GmbH, Oberkochen, Germany). Pictures were taken with a digital Axiocam camera system (Carl Zeiss GmbH; model CF20DXC). Analysis was restricted to decidual extravillous tissues due to the almost exclusive abundance of HLA-G-expressing cells in these tissues.

### Statistical analysis

Groups were compared using non-parametrical Mann–Whitney *U*-signed rank tests or Kruskal–Wallis’s test as appropriate. Multiple linear regression models were used to analyse the associations of clinical characteristics (body mass index [BMI] and birthweight) with IRS. The SPSS statistical package version 22.0. for Windows (IBM Corp., Armonk, NY, USA) was used for data collection, analysis and visualization. Statistical significance was accepted at *p* values < 0.05.

## Results and discussion

**Fig. 1:**
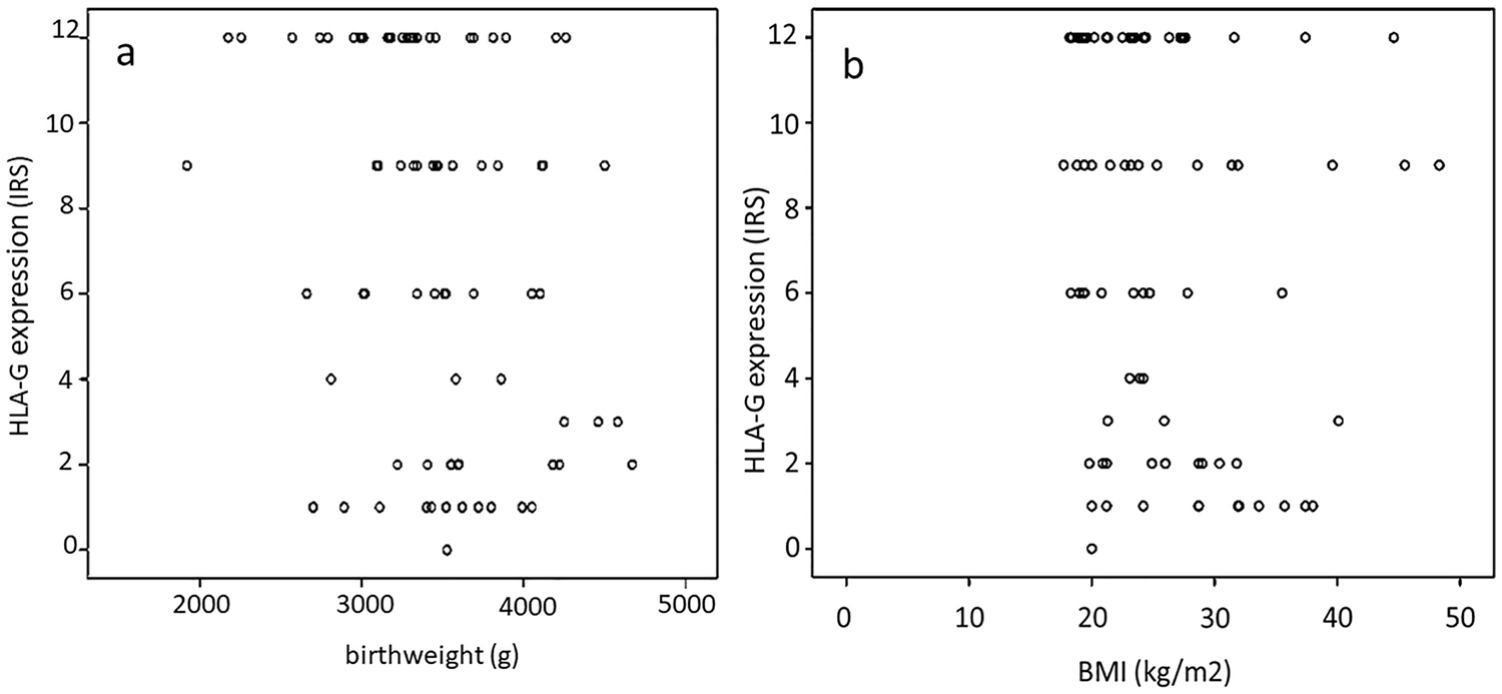
Correlation of clinical characteristics (birthweight and pre-pregnancy BMI) with immunofluorescence staining. Neither birthweight (g) (**a**) nor BMI (kg/m^2^) (**b**) correlated with the HLA-G IRS.* BMI* Body mass index, *HLG-G* human leucocyte antigen G,* IRS* immunoreactivity score,

The study cohort was fully described in earlier studies on changes in the expression of nuclear receptors associated with GDM (Knabl et al. 2020, 2015a, b). A major strength of the present study is the objectively assessed absence of GDM in the control group: all women participating in the study underwent an oGTT. Women with GDM had higher pre-pregnancy BMI and their newborn had a higher birthweight than the non-GDM controls (Table 1). Multiple linear regression analysis was used to assess potential confounding or interaction with HLA-G IRS, which showed that neither birthweight nor pre-pregnancy BMI were associated with HLA-G IRS (*p* > 0.05) (see Fig. 1a, b).

Analysis of GDM and control subgroups did not reveal any correlation with HLA-G staining, apart from a negative correlation with birthweight in the male control group (correlation coefficient − 0.63; *p* < 0.05). Although this result is of general interest, it does not interfere with our specific finding of reduced HLA-G staining between the GDM and control groups.

We first aimed to test for potential changes in the cellular HLA-G location in the GDM group. HLA-G double fluorescence staining was used to discriminate between total trophoblast tissue (CK7 positive) and HLA-G. The antibody used was specific for isotypes HLA-G1 and HLA-G5, both regarded the most biologically relevant isotypes (Bainbridge et al. 2000).

**Fig. 2:**
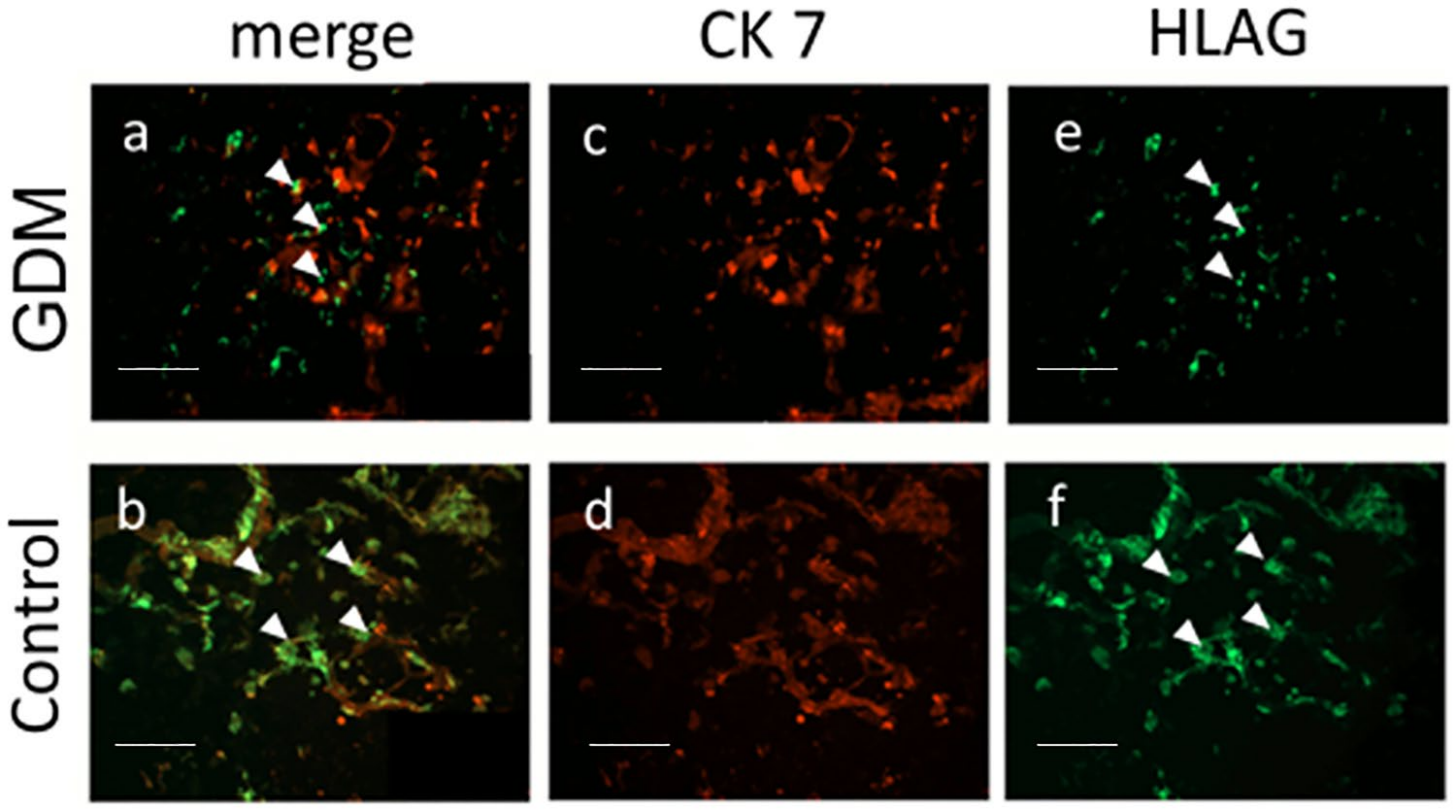
Phenotyping of extravillous tissue by double immunofluorescence staining.** a**,** b** Triple filter excitation showing both CK7 and HLA-G. **b**, **c** CK7, bound by Cy-3-labelled secondary antibody, stained red, marking extravillous trophoblasts (EVTs) and syncytiotrophoblast (SCN) together. **e**, **f** HLA-G, bound by Cy-2-labelled secondary antibody, stained green, marking the EVT. HLA-G labelling can be seen to be reduced in the GDM group (**a**) compared to the control (**b**) in EVT. Arrowheads indicate HLA-G-expressing cells Scale bar: 100 µm.* GDM* Gestational diabetes mellitus

In Figure 2 HLA-G-specific-staining in the decidua of placentas of women with GDM is shown in green, while CK7 expression is shown in red. In both the GDM and control groups we identified HLA-G on the cell surface, indicating that only membrane bound HLA-G was visualized as soluble HLA-G cannot be demonstrated by immunohistochemistry. In normal control decidual tissue, EVTs were labelled evenly, with HLA-G in green (Fig. 2e, f) and in general with CK7 in red (Fig. 2 c, d). Triple filter excitation (Fig. 2a, b) showed a stable co-expression of HLA-G (EVTs) and CK7. Visual impression was that there was a reduction of HLA-G expression on the cell surface in EVTs of the GDM EVT as compared to the non-GDM controls (compare Fig. 2e, f).

To substantiate this observation, we used semi-quantitative immunohistochemistry with an antibody that reacts with all isoforms. Staining intensity was semi-quantified in a cell type-specific manner by calculating the IRS, which is a measure that integrates the number of stained cells with staining intensity and allows overall proteins levels to be semi-quantified in tissues in a cell type-specific manner. This technique has been successfully used in earlier studies (Knabl et al. 2015a, b, 2020).

**Fig. 3:**
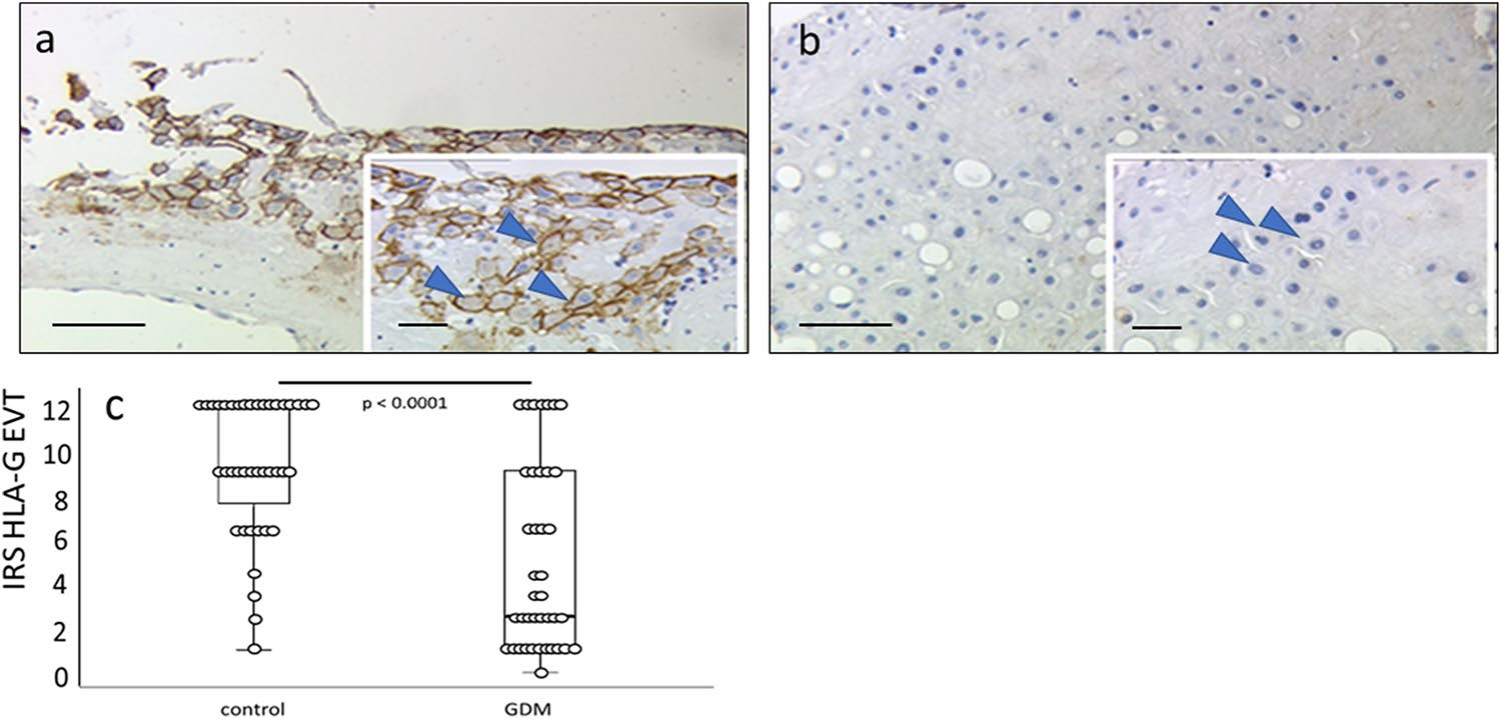
Immunoreactivity of EVTs in the GDM and control groups.** a**,** b** Strong HLA-G immunoreactivity was found in control EVTs (**a**) and significantly weaker EVT immunolabelling was observed in the GDM group (*p* < 0.001), Scale bar: 100 μm, scale bar of insert: 50 μm. (**b**). Arrowheads in insets indicate EVT cells with different immunoreactivity. Scale bar: 100 μm, scale bar of insert: 50 μm** c** IRS for each group is shown as box plots for EVT (IRS HLA-G EVT). The boxes represent the range between the 25th and 75th percentiles, the horizontal line represents the median, bars represent the 5th to 95th percentiles and open circles represent single values in each group

The IRS for HLA-G immunolabelling was reduced by 66% in the EVTs of the placenta of women with GDN versus the non-GDM controls (*p* < 0.0001; median IRS: 3 [GDM group vs. 9 [control group]) (Fig. 3). Importantly, we could demonstrate that this downregulation is not due to a loss of cell number, but to a loss of protein expression on EVTs in general because CK7 expression was found to be equally distributed on the EVTs of both the GDM and controls groups.

For confirmation of our results, we performed an analysis of CK7 expression in double immunofluorescence-stained sections in a semi-quantified manner. Eight cells per group (GDM vs. control) were analysed, comparable to the number used to determine the IRS. No significant differences (*p* = 0.87, Kruskal–Wallis test) were found.

**Fig. 4:**
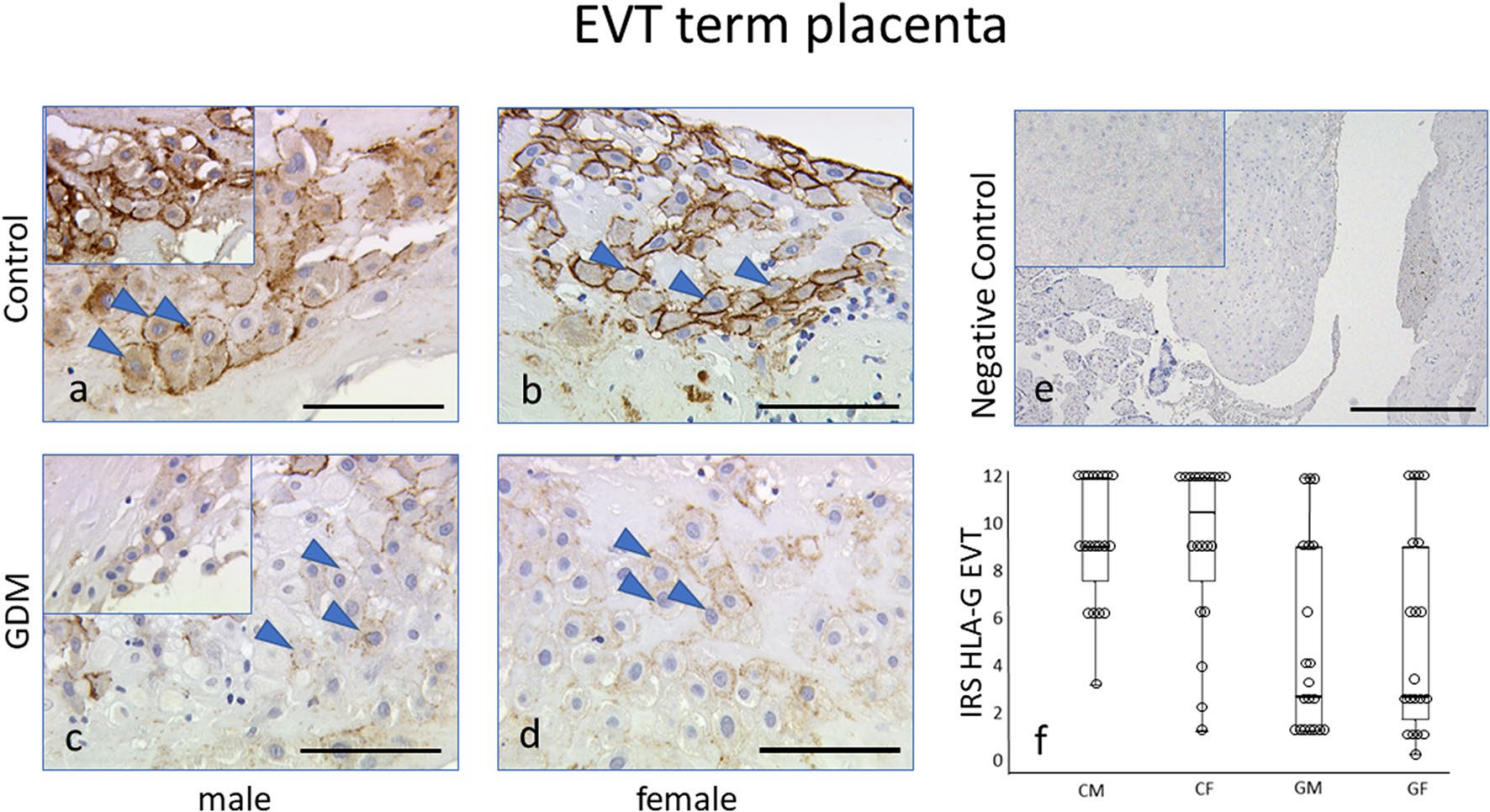
Immunoreactivity of EVTs in the GDM and control groups after stratification for fetal sex.** a**,** b** HLA-G immunoreactivity was found at a similar intensity in EVTs in the male control subgroup (**a**) and in the female control subgroup (**b**).** c**,** d**, In the GDM group, similarly, no gender differences were identified between the male GDM subgroup (**c**) and the female GDM subgroup (**d**). **e** Negative control in term placenta.** f** IRS for each group is shown as box plots for EVTs (IRS HLA-G EVT). Boxes represent the range between the 25th and 75th percentiles, the horizontal line represents the median, bars represent the 5th to 95th percentiles and open circles represent single values in each subgroup. Arrowheads indicate EVT cells. **a–d** Scale bar: 100 μm, **e** Scale bar: 200 μm. *CM* Control male subgroup, *CF* control female subgroup, *GM* GDM male subgroup, *GF* GDM female subgroup

Since sex-specific differences are common in placental function and the prevalence of pregnancy disorders as well as fetal outcome differ between fetal sex, we analysed the potential effect of fetal sex (Table 2 for clinical details stratified by fetal sex). Again, women with GDM had higher pre-pregnancy BMI and their newborns had a higher birthweight than their non-GDM controls—importantly in both male and female offspring. Multiple linear regression analysis was used to assess potential confounding factors, revealing that neither birthweight nor BMI were potential confounders associated with HLA-G IRS in the placentas of both male and female offspring (see Fig. 1a, b). No sex-differences in the GDM-associated reduction of HLA-G IRS was observed. After stratification for fetal sex, immunostaining showed no sex-specific difference within the controls group (mean IRS: 9.0 [male] vs. 10.5 [female]; *p* > 0.05), nor within GDM (mean IRS: 2.0 [male] vs. 2.0 [female]; *p* > 0.05) (Fig. 4).

The biological consequences of less HLA-G in the GDM group versus the non-GDM controls are unclear. Placental inflammation is often found in pregnancies complicated by GDM (Kleiblova et al. 2010), with the hyperglycemia producing an inflammatory environment with a high content of inflammatory cytokines and immune cells. The number of NK cells is increased especially in the extravillous layer of the human placenta in women with GDM and type 2 diabetes mellitus (Hara et al. 2016). NK cells are immune cells with close contact to EVT cells and are found abundantly in the uterine decidua. NK cells generally destroy cells which do not express HLA class-la molecules, but HLA-G protects the extravillous cells through cytolysis of NK cells (Rajagopalan and Long 1999). Therefore, our data could provide a possible link between the enhanced inflammation often associated with GDM and an altered immune response by a consequent loss of HLA-G expression (Sharma et al. 2021).

Secondly, HLA-G-specific interactions contribute to placental development through the secretion of angiogenic and pro-inflammatory factors by decidual NK cells and macrophages (Li et al. 2009). Therefore, HLA-G may be implicated in vascular remodelling and apoptosis of damaged cells, which is an important process that regulates the extent of trophoblast invasion (Rouas-Freiss et al. 2021). Whether GDM is associated with altered trophoblast invasion remains to be studied.

## Summary and conclusion

The results of this study show that GDM did not change the location of cell-surface HLA-G in term placentas, as reflected by similar staining patterns in the GDM group and the non-GDM control group with HLA-G immunoreactivity restricted to EVTs. For the control group, this result is in line with those of an earlier study on term placentas from women with uncomplicated pregnancies (O'Callaghan and Bell 1998). However, the protein levels of HLA-G, as reflected by IRS, were reduced in EVTs of the GDM group as compared to the non-GDM control group. An important strength of our study is the objective assessment by oGTT of GDM absence in the control group. While potential confounders such as pre-pregnancy BMI and birthweight were considered in our analysis, residual confounding cannot be excluded.

In conclusion, our study identified a specific downregulation of HLA-G in the EVT cells of term placentas of pregnant women with GDM that is not caused by cell loss. These results may provide new knowledge about the changes in cellular immunoregulation at the materno-fetal interface in pregnant women with GDM. Whether these changes are specific for GDM or are a general phenomenon of all forms of diabetes mellitus, i.e. also seen in type 1 and type 2 diabetes mellitus, remains to be studied.

## Abbreviations

BMI: Body mass index
EVT: Extravillous trophoblast
GDM: Gestational diabetes mellitus
HLA: Human leucocyte antigen
IRS: Immunoreactivity score
SCT: Syncytiotrophoblast
